# Passive knee exoskeletons in functional tasks: Biomechanical effects of a *SpringExo* coil-spring on squats

**DOI:** 10.1017/wtc.2021.6

**Published:** 2021-06-08

**Authors:** Rand Hidayah, Dongbao Sui, Kennedi A. Wade, Biing-Chwen Chang, Sunil Agrawal

**Affiliations:** 1 Department of Mechanical Engineering, Columbia University, New York City, New York, USA; 2 State Key Laboratory of Robotics and System, Harbin Institute of Technology, Harbin, China

**Keywords:** biomechanics, design, exoskeletons, performance augmentation, performance characterization

## Abstract

Passive wearable exoskeletons are desirable as they can provide assistance during user movements while still maintaining a simple and low-profile design. These can be useful in industrial tasks where an ergonomic device could aid in load lifting without inconveniencing them and reducing fatigue and stress in the lower limbs. The *SpringExo* is a coil-spring design that aids in knee extension. In this paper, we describe the muscle activation of the knee flexors and extensors from seven healthy participants during repeated squats. The outcome measures are the timings of the key events during squat, flexion angle, muscle activation of rectus femoris and bicep femoris, and foot pressure characteristics of the participants. These outcome measures assess the possible effects of the device during lifting operations where reduced effort in the muscles is desired during ascent phase of the squat, without changing the knee and foot kinematics. The results show that the *SpringExo* significantly decreased rectus femoris activation during ascent (−2%) without significantly affecting either the bicep femoris or rectus femoris muscle activations in descent. This implies that the user could perform a descent without added effort and ascent with reduced effort. The exoskeleton showed other effects on the biomechanics of the user, increasing average squat time (+0.02 s) and maximum squat time (+0.1 s), and decreasing average knee flexion angle (−4°). The exoskeleton has no effect on foot loading or placement, that is, the user did not have to revise their stance while using the device.

## Introduction

**Figure 1.**
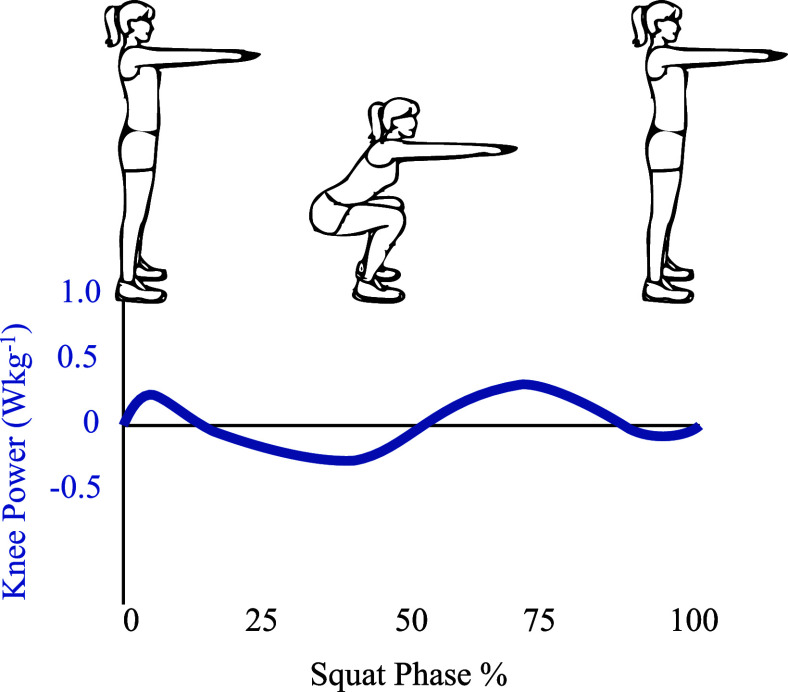
The joint power profiles of the knee joint during a typical squat motion, adapted from Hwang et al. (2009). The upright positions show the beginning (0%) and end (100%) of the squat cycle. The lowest point of a squat occurs at 50% of the squat cycle. The knee power profile exhibits a clear shift from negative to positive before and after the 50% point. The negative power due to gravitation assistance of the descent can be stored as elastic energy in a spring. The spring can be designed such that the stored energy is released in ascent to assist knee extension. This is the principle behind the *SpringExo* design used in the study presented in this paper.

Passive exoskeletons use the biomechanics of human motion to improve the efficiency of performing the tasks (Carrozza et al., 2017). The advantage of passive wearables is their low profile, allowing their use in everyday life without inconveniencing the user (Carrozza et al., 2017). These characteristics suit industrial environments where reducing effort and joint effort is desirable, especially in lifting tasks. The knee joint power and moment profiles of a lifting task, characterized by Hwang et al. (2009), show distinct phases of work done on (or by) the knee joint, as seen in Figure 1. Commercial passive exoskeletons typically assist in maintaining specific postures for extended amounts of time, such as the Chairless Chair (Noone, Deizisau, Germany) for long-standing tasks, the ShoulderX (US Bionics, Berkeley, CA), the Paexo (Ottobock, Duderstadt, Germany) for raised arm tasks, and the ZeroG (Eksobionics, Richmond, VA) for tool carrying. These exoskeletons provide support in a static position but cannot support the dynamic task of lifting during squatting. This task requires several muscles to stabilize the body, as shown by Escamilla et al. (2001). The body is stabilized in the dynamic squatting task by the vastus lateralis, vastus medialis, and gluteus maximus, and extending the knee in ascent recruits the rectus femoris and biceps femoris muscles to provide strength for lifting.

In the literature, Van Dijk et al. (2011) proposed the design methodology of using the phase of a task where energy is stored when work is done on a joint and energy is returned when work is done by a joint. This was the methodology used by the passive knee-ankle exoskeleton targeting the storage and return of energy during distinct phases of the gait cycle by Etenzi et al. (2020). The mechanics and energetics of gait using unpowered ankle exoskeletons were characterized by Sawicki and Ferris (2008), and this characterization informs designs of ankle exoskeletons for further studies in the literature. The ankle designs have targeted optimal stiffness for metabolic cost reduction in gait and running, as shown by the work of Collins et al. (2015) and Nasiri et al. (2018). Witte et al. (2020) proposed a powered and unpowered exoskeleton and studied the effects on human running. Passive ankle exoskeletons have been designed with the goal of widespread adoption (Yandell et al., 2019).

Other passive lower limb exoskeletons focus on the hip joint and target net energetic decreases during walking by the use of an elastic band (Haufe et al., 2020), maintaining balance while walking (Zhang et al., 2018), and increasing walking distance for neurological patients (Panizzolo et al., 2021).

An industrial worker typically lifts a load while stooping or performing a squat, with the latter being preferable as it recruits more muscles from the lower limbs (Hwang et al., 2009). In this paper, we focus on squatting. During squat ascent, the rectus femoris acts as a biarticular hip flexor and knee extensor. These effects are more pronounced when the trunk is upright, as noted by Escamilla (2001). The stance and placement of the feet in a squat do not affect activations of muscles at the knee (Escamilla, 2001) but affect the knee kinetics, as shown by Almosnino et al. (2013) and Lorenzetti et al. (2018). Ideally, a device that aids in reducing muscle activation should not significantly affect the user’s preferred stance and foot loading profiles.

**Figure 2.**
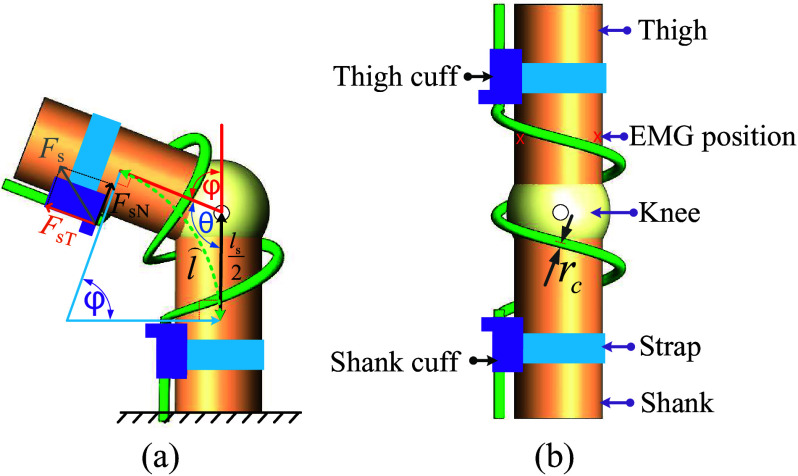
A kinematic diagram of the *SpringExo* donned on the leg. The leg is flexed at an angle *φ* with *SpringExo* donned (a) and the resulting Force *F_s_* that can be decomposed into the normal and tangential components *F_sT_* and *F_nT_.* The original state of the upright leg with the *SpringExo* donned (b) with the mechanical components that make up the *SpringExo.*

We propose a passive, unpowered exoskeleton *SpringExo* to assist knee extension and reduce the rectus femoris activation during ascent in a squatting task. The *SpringExo* design (Figure 2) uses the power profile shown in Figure 1 to store energy in descent and returns it during ascent. The *SpringExo* can store elastic energy while the user descends. If the spring can contribute to the joint torque in ascent, we can expect less effort expended by the user and decrease muscle activation in ascent. We investigate whether this effect is present with a *SpringExo* worn around each leg with seven healthy subjects in this paper. We use the methodology proposed by Van Dijk et al. (2011) to explore the feasibility of decreasing the rectus femoris activation in a squatting task. This paper aims to characterize the change in muscle activation, mainly focusing on the rectus femoris as the knee extensor that is activated during ascent, as found by Escamilla et al. (2001) during squats. The rectus femoris is also the primary muscle activity decrease targeted during squat lifting, such as the motorized quasi-passive device in Yu et al. (2019) and the entirely passive torsional spring design in Ranaweera et al. (2018).

The *SpringExo* is an entirely passive system that targets knee extension while remaining low profile to wear under a user’s clothing. It does not require a motor, unlike the device presented in Yu et al. (2019), and requires fewer components than the passive exoskeleton in Ranaweera et al. (2018). The simplicity of the design and incorporating the spring elements into the architecture itself are the main features that make the *SpringExo* a novel wearable device, similar to the ankle exoskeleton presented in Yandell et al. (2019).

We test whether the *SpringExo* reduces rectus femoris activation in a squatting task and characterize the effects on the user’s squat biomechanics. Primary measures include the timing, knee flexion angles, and the rectus femoris and biceps femoris muscle activations. In addition, we also verify that our device does not affect the user’s foot loading preference by looking at the exoskeleton’s effect on the distance between pressure maximums at the ball and heel of each user’s feet and the total pressure loading line lengths on the right and left feet.

## Methodology

### Design of SpringExo

**Figure 3.**
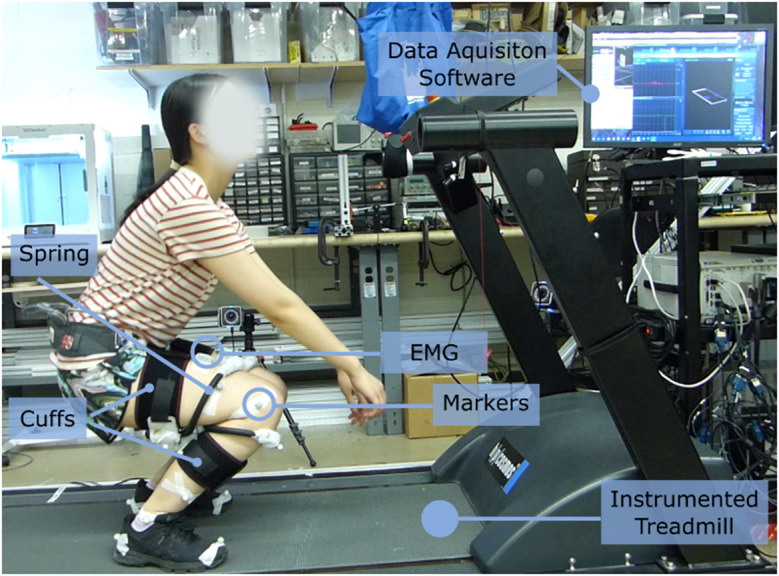
A user performing a squat in the *SpringExo* passive exoskeleton. The instrumented treadmill is used to collect foot pressure and loading data. The motion of the human body is captured using infrared markers. Surface EMG signals are used to collect muscle activation data.

The passive *SpringExo* in Figure 2 is a 3D-printed nylon spring coil attached to a photopolymer resin 3D-printed thigh cuff and shank cuff, which can strap to the thigh and shank of the user. The coil spring can accommodate extreme knee flexion in squat kinematics, such as in Tewart (2012). The cuffs are mechanically connected to the spring ends and attached to the leg via Velcro straps covering the skin to avoid discomfort. The coils do not collide when a user performs a squat as they both revolve in the same direction. The complete assembly weighs 450 g. The exoskeleton is easily donned and doffed while securing the leg. A user wearing the *SpringExo* is shown in Figure 3. The parts and instrumented treadmill used in this study are labeled.

The *SpringExo* only assists in knee extension during the ascent part of the squat cycle while the energy is stored during descent. The spring bending angle 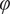 compresses the spring and results in the Force 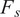 acting on the thigh. The angle 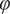 is the central spring angle defined as:
(1)

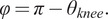




The spring exerts a force 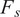 on the leg due to the bending arc of the spring. The net force is composed of a tangential component 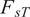 that supports the leg, and a normal component 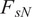 that provides assistance in leg extension, as shown in Figure 2.
(2)

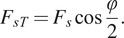



(3)

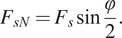




The Spring mechanism and stored potential energy of this device is described in Sui et al. (2021). We briefly present this mechanism here. The total elastic potential energy 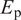 stored in the spring comes from compression 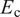 and bending 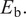



(4)

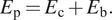




In compression, the virtual center length of the spring changes from its free length 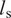 to the arc length 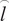 , and the elastic potential energy 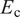 stored in the spring with a stiffness of 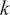 is:
(5)

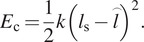




In bending, according to Kim et al. (2017), Gutkowski (1998), and Wahl (1963), the total stored potential energy 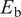 due to the spring bending with a constant arc length 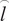 is expressed as:
(6)

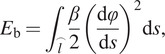



(7)

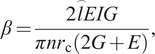

where 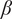 , 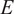 , 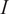 , 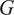 , 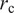 , 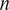 , and 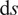 are, respectively, the flexural rigidity of the spring, Young’s modulus, area moment of inertia, shear modulus, mean radius of the spring coil, number of the spring coil, and element of the arc length 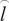 . According to equations (4)–(7), 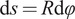 , and the geometric relationship in Figure 2a, the total stored elastic potential energy 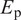 is expressed as:
(8)

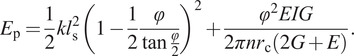




The *SpringExo* has an outer diameter 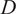 = 167 mm, a coil diameter of 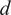 = 16.5 mm. Nylon-66’s has a Flexural Modulus 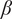 of 2,700 Nmm^−2^, and Young’s modulus 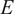 of 3.7 Gpa. The analysis and optimization of these parameters for *SpringExo* stiffness are not included in this work.

### Subjects

A convenience sample of seven healthy subjects (four females)—age 25.9 ± 2.2 years, height 167.6 cm ± 4.6 cm, and mass 54.3 kg ± 5.8 kg—were recruited to carry out the study. Investigators collected informed consent from subjects in the study approved by the Institutional Review Board of Columbia University.

### Experimental Setup and Protocol

Subjects performed squatting motions on an instrumented treadmill (Pluto, HP Cosmos, Nußdorf, Germany) with an integrated pressure sensor to measure foot pressure at a frequency of 100 Hz. Markers were placed on the distal phalanges, ankle, heel, tibia, knee, femur, and trochanter to capture knee motion data at 200 Hz (Vero, Vicon, Oxford, United Kingdom). Wireless EMGs were placed on the rectus femoris and bicep femoris muscles. These data were captured at 2,000 Hz (Trigno, Delsys, Cambridge, MA) in the marked positions shown in Figure 3.

A baseline (*B*) condition where the subjects performed squats wearing no device was compared against the condition where the *SpringExo* exoskeleton was worn (*E*). Subjects completed squat cycles at their own pace in each condition. Subjects were assigned either the *B* or *E* condition in a random order. Each measurement started and ended with the subjects standing neutrally and at rest. Subjects did not acclimatize to the springs by practicing with the design and performed the squats directly after donning the device. Investigators instructed subjects to squat continuously until tired. The first five squat cycles were discarded. The next 20 cycles from each subject were analyzed and interpreted.

### Data Processing

All data preparation and processing was done using Python 3.6 (Python, Wilmington, DE). Kinematics of the knee joint was solved using the marker set on the leg. Markers attached to the hip were used to segment each squat cycle, where the upright position was used as an endpoint. Each segment’s EMG data and joint angles from both legs were normalized into a cycle, where 0% indicates the initial standing position, 25% indicated halfway through descent, 50% indicates the lowest position, 75% indicates halfway through ascent, and 100% indicates the terminal standing position. A fourth-order band-pass (20–400 Hz) Butterworth filter was used to filter the raw EMG signals, rectified and passed through another fourth-order low-pass (3 Hz) Butterworth filter. Each subject’s cycles were *B*-normalized using the subject’s maximum muscle activation values in *B* sessions.

Each subject’s EMG signals were normalized by their respective *B* maximum values. All subjects’ knee flexion angles, normalized rectus femoris (nRF), and normalized bicep femoris profiles were averaged across both legs. These profiles were used to calculate averages, maxima, and the descent and ascent values for each parameter. The knee kinematics and muscle activations of each mode compare the effect of the worn *SpringExo* on user biomechanics during a squat exercise.

The foot pressure maximum at the ball and the heel of the foot were found for each cycle. The loading line length and stance width of the feet were also extracted across the same cycles. Differences in these parameters between the two conditions indicate the effects of wearing the *SpringExo* on the user’s overall foot placement patterns. Differences in overall foot loading and placement indicate changes that can adversely affect the person’s safety and comfort in performing the squatting task.

### Statistical Analysis

The mean values, maximum values, minimum values, values at 25% and 75% of the squat cycle, and their standard deviations to the mean were calculated for knee flexion, muscle activations, and foot loading parameters. Each cycle’s values for each subject were grouped according to the mode *B* or *E.* The data were tested for normality using Kolmogorov–Smirnov one-sample tests. The data violated normality, and so nonparametric tests were selected to compare the conditions. A Mann–Whitney *U* test was used to compare the values reported in this paper. Significance was set at 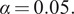


## Results

**Figure 4.**
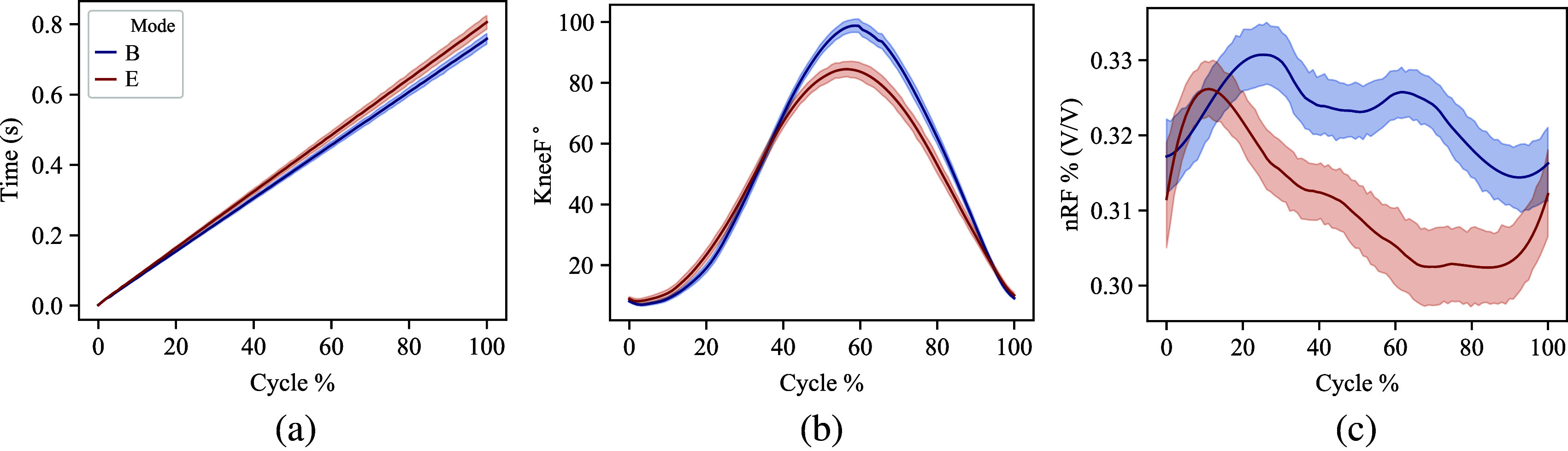
The averaged user behavior in *B* and *E* modes across all subjects and squat cycles. All data were segmented into squat cycles and normalized to the same cycle percentage. 4a represents the timing information of each mode. 4b shows the knee flexion angles in each mode. 4c shows the muscle activation magnitude of the rectus femoris in each mode, normalized in magnitude to the maximum value in *B.*

Figure 4 shows the changes in the subjects’ squat cycle timing, knee flexion profiles, and muscle activation due to the *B* or *E* mode. We summarize the observations qualitatively here with values reported further in this section—an increase in overall squat cycles’ timing is observed. The maximum flexion angle of the knee is smaller when wearing the exoskeleton when compared to the baseline. The rectus femoris muscle activity is lower, particularly in the ascent phase of the squat.

**Figure 5.**
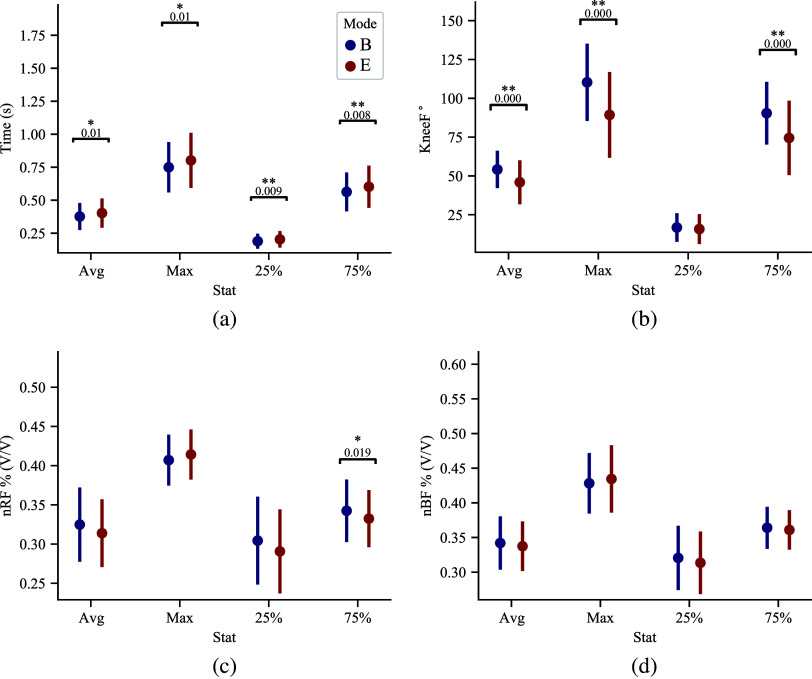
Extracted averages and standard deviations, maximums, minimums, and values at 25% of the squat cycle and 75% of the squat cycle for each variable. These parameters were extracted from Figure 4 data and used to compare user behavior for the *B* and *E* modes. 5a shows the timing of each squat, 5b shows the knee flexion angle parameters, 5c shows *B*-normalized rectus femoris muscle activity, and 5d shows *B*-normalized bicep femoris muscle activity. The results of the Mann–Whitney *U* test are shown in the subfigures as *p*-values and with a * when *p* < .05 and a ** when *p* < .01.

**Figure 6.**
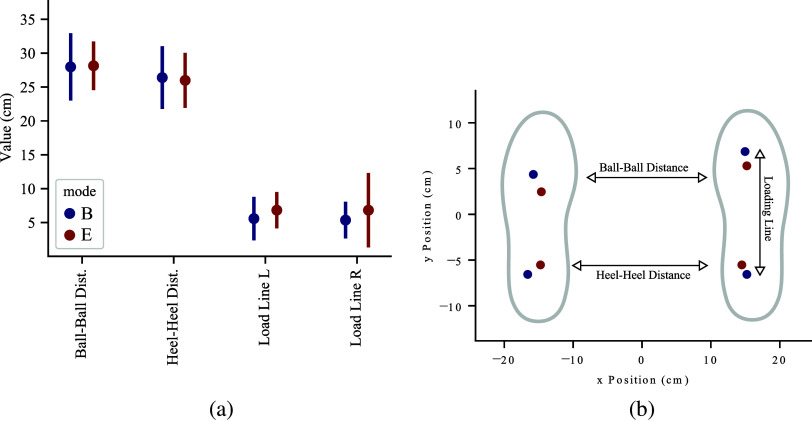
Foot loading behavior of the user with the exoskeleton donned and doffed. 6a shows the effect between each mode, *B* and *E* on the loading of the feet across all subjects. The variables shown are the distances between the balls and heels of the right and left feet, as well as the length of the pressure loading lines of each foot. 6b shows a visual representation of these variables.

Figure 5 shows timing, knee flexion, and muscle activity averages, maximums, and the descent (25%) and ascent (75%) points of the squat cycle. Figure 6 shows the effects of wearing the passive exoskeleton on specific intervals of the squat cycle across all subjects and exoskeleton modes.

The differences are characterized and compared statistically in Figure 5. Significant differences from *B* to *E* are summarized in Figure 5 with *p*-values reported. We see an increase in squat cycle timing overall between *B* and *E* (Avg: 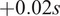 , Max: 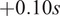 , 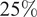 : 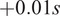 , 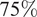 : 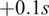 ). We see a decrease in nRF activation in ascent between *B* and *E* ( 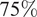 : 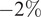 ). No statistically significant difference is observed in the activation of bicep femoris during the gait cycle. An overall decrease in knee flexion angles is observed (Avg: 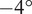 , Max: 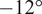 , 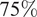 : 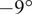 ).

Figure 6 shows the effects of wearing the exoskeleton on the foot loading parameters across all subjects in both modes. Figure 6b defines the foot loading parameters discussed in this paper. There were no significant differences observed between *B* and *E* modes for the ball–ball or heel–heel distances of the feet. No significant difference was observed for the loading lines of the left or right foot either. No other significant changes were observed. The subjects have a smaller range of foot loading parameters in *E*, except for loading on the right leg, which increases in range, as shown in Figure 6a. The statistical analysis yielded marginal *p*-values for ball–ball distance ( 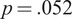 ). The ball–ball distance increased slightly on average ( 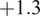 cm), which indicates that frontal foot pressure moved away from the midline. More subjects are required to verify a conclusive effect of wearing the *SpringExo* device.

## Discussion

The *SpringExo* was able to significantly decrease RF activation during ascent, roughly at 75% of the squat cycle. The device did not significantly increase muscle activation in any other phase of the cycle. The device did not affect the BF muscle activity. The decrease in muscle activation indicates that a simple coil spring architecture is a suitable ergonomic choice in this specific movement in an industrial context, where muscle activity reduction is desirable. Other user effects include a longer squat cycle time, indicating that the device affected the user’s chosen velocity for performing a squat task. A significant decrease in knee flexion angle was seen.

The restriction of the knee angle is a limitation that must be addressed by testing different spring stiffness that produces a reduction in EMG values while still retaining the same range of motion of flexion in the knee at the halfway point of the squat cycle. This can be done by following the methodology for empirically testing for optimal spring stiffness and the effects on EMG activation in a study similar to the one done in Collins et al. (2015). An optimal spring stiffness for a specific cohort of users can then be determined by having users complete the same squatting task with the same knee flexion magnitudes and evaluating the muscle activation decrease. Furthermore, the increase in the timing of performing the squat task could contribute to the lower muscle activation of the rectus femoris, as the velocity of doing a squat has an effect on the activation and recruitment of muscle groups in the quadriceps as characterized by Stasinaki et al. (2019). Our study aimed to characterize the effect on the primary muscle that aids in knee extension, and effects on the stabilizing muscles of gluteus maximus, vastus lateralis, and vastus medialis were not investigated. This is a potential area of investigation for a study that fully characterizes the effect of *SpringExo* in a study looking at loaded lifts, the effect of spring stiffness, or adding a motor.

The foot loading parameters show that donning the *SpringExo* did not significantly change the users’ stance. Foot placement in a dynamic squat task can significantly change the person’s lower limb kinetics performing the task (Almosnino et al., 2013). For example, Demers et al. (2018) showed that narrower stances in squats could indicate larger hip adduction torques. The foot loading parameters do not significantly change with the worn device. This lack of change implies that the device does not impede or interfere with the user’s foot loading preferences while squatting. This points to this device’s suitability in an industrial workspace, as it would not interfere with comfort or preferences of a user while descending and ascending from a bodyweight squat. It is a limitation of this study that a loaded lifting task was not considered, as the foot loading preferences after picking up a load would be more informative for the real-world application with this device. We can expand the device’s capabilities to target assistive lifting tasks such as in Yu et al. (2019), where the motor’s torque magnitude was used to reduce RF activation to different levels. Hwang et al. (2009) characterized the magnitude of the power required for squat ascent at the knee, which can be used to select an appropriate motor to compensate for part or most of the biological energy necessary for the lifting task.

Future investigations that can be carried out with this device include the exploration of motorizing this system such that a quasi-passive mode can be used to aid in descent and release the stored energy in ascent. The *SpringExo* would ideally be used on its own in a passive mode to reduce parts and complexity, and as-needed can be motorized to optimize performance, as shown in Sui et al. (2021).

## Conclusion

We designed a passive exoskeleton that targeted storing elastic energy during squat descent and returning this energy to the squat ascent phase. The spring exoskeleton reduced muscle activation in the rectus femoris during ascent in the squat cycle without increasing bicep femoris activation. The knee flexion angle decreased overall but without significant changes in foot loading and placement parameters. Future investigation into different spring stiffness or a motor to aid in descent is required to optimize utility from this simple and easily wearable device.

## Data Availability

Data can be made available to interested researchers upon request by email to the corresponding author.

## References

[r1] Almosnino S , Kingston D and Graham RB (2013) Three-dimensional knee joint moments during performance of the bodyweight squat: Effects of stance width and foot rotation. Journal of Applied Biomechanics 29(1), 33–43. 23462440 10.1123/jab.29.1.33

[r2] Carrozza M , Silvestro C , Jos M , Symposium I and Robotics W (2017) Wearable robotics: Challenges and trends. In: Dino Accoto , Sunil Agrawal , Fabio Babiloni , Jose M. Carmena , Maria Chiara Carrozza , Paolo Dario , Arturo Forner-Cordero , Masakatsu G. Fujie , Nicolas Garcia , Neville Hogan , Hermano Igo Krebs , Dirk Lefeber , Marko Munih , Paolo M. Rossini , Atsuo Takanishi , Russell H. Taylor , David A. Weitz , Loredana Zollo eds. Proceedings of the 2nd International Symposium on Wearable Robotics, vol. 16 219–257. Segovia: Springer.

[r3] Collins SH , Bruce Wiggin M and Sawicki GS (2015) Reducing the energy cost of human walking using an unpowered exoskeleton. Nature 522(7555), 212–215. 25830889 10.1038/nature14288 PMC4481882

[r4] Demers E , Pendenza J , Radevich V and Preuss R (2018) The effect of stance width and anthropometrics on joint range of motion in the lower extremities during a back squat. International Journal of Exercise Science 11(1), 764–775. 29997725 10.70252/BWZE8275 PMC6033510

[r5] Escamilla RF (2001) Knee biomechanics of the dynamic squat exercise. Medicine and Science in Sports and Exercise 33(1), 127–141. 11194098 10.1097/00005768-200101000-00020

[r6] Escamilla RF , Fleisig GS , Lowry TM , Barrentine SW and Andrews JR (2001) A three-dimensional biomechanical analysis of the squat during varying stance widths. Medicine and Science in Sports and Exercise 33(6), 984–998. 11404665 10.1097/00005768-200106000-00019

[r7] Etenzi E , Borzuola R and Grabowski AM (2020) Passive-elastic knee-ankle exoskeleton reduces the metabolic cost of walking. Journal of Neuroengineering and Rehabilitation 17(1), 104. 32718344 10.1186/s12984-020-00719-w PMC7385868

[r8] Gutkowski W (1998) Advanced mechanics of structures. European Journal of Mechanics—A/Solids 17(6), 1044–1045.

[r9] Haufe FL , Wolf P , Riener R and Grimmer M (2020) Biomechanical effects of passive hip springs during walking. Journal of Biomechanics 98, 109432. 31662197 10.1016/j.jbiomech.2019.109432

[r10] Hwang S , Kim Y and Kim Y (2009) Lower extremity joint kinetics and lumbar curvature during squat and stoop lifting. BMC Musculoskeletal Disorders 10(1), 15. 19183507 10.1186/1471-2474-10-15 PMC2651112

[r11] Kim Y , Cheng SS , Diakite M , Gullapalli RP , Simard JM and Desai JP (2017) Toward the development of a flexible mesoscale MRI-compatible neurosurgical continuum robot. IEEE Transactions on Robotics 33(6), 1386–1397. 29225557 10.1109/TRO.2017.2719035 PMC5718214

[r12] Lorenzetti S , Ostermann M , Zeidler F , Zimmer P , Jentsch L , List R , Taylor WR and Schellenberg F (2018) How to squat? Effects of various stance widths, foot placement angles and level of experience on knee, hip and trunk motion and loading. BMC Sports Science, Medicine and Rehabilitation 10(1), 14. 10.1186/s13102-018-0103-7 PMC6050697 30026952

[r13] Nasiri R , Ahmadi A and Ahmadabadi MN (2018) Reducing the energy cost of human running using an unpowered exoskeleton. IEEE Transactions on Neural Systems and Rehabilitation Engineering 26(10), 2026–2032. 30281466 10.1109/TNSRE.2018.2872889

[r14] Panizzolo F , Cimino S , Pettenello E , Belfiore A , Petrone N and Marcolin G (2021) Effect of a passive hip exoskeleton on walking distance in neurological patients. Assistive Technology, 1–6. 10.1080/10400435.2021.1880494. 33481693

[r15] Ranaweera R , Gopura R , Jayawardena T and Mann G (2018) Development of a passively powered knee exoskeleton for squat lifting. Journal of Robotics, Networking and Artificial Life 5(1), 45.

[r16] Sawicki GS and Ferris DP (2008) Mechanics and energetics of level walking with powered ankle exoskeletons. Journal of Experimental Biology 211(9), 1402–1413. 18424674 10.1242/jeb.009241

[r17] Stasinaki A-N , Zaras N , Methenitis S , Bogdanis G and Terzis G (2019) Rate of force development and muscle architecture after fast and slow velocity eccentric training. Sports (Basel) 7(2), 41. 30769873 10.3390/sports7020041 PMC6410101

[r18] Sui D , Chang BC , Hidayah R , Zhu Y and Agrawal S (2021) SpringExo, a spring-based exoskeleton for providing knee assistance: Design, characterization and feasibility study. In IEEE International Conference on Robotics and Automation (ICRA). Xi’an, China.

[r19] Tewart ARDS (2012) A biomechanical comparison of the traditional squat, powerlifting squat and box squat. Journal of Strength and Conditioning Research 26(7), 1805–1816. 22505136 10.1519/JSC.0b013e3182577067

[r20] Van Dijk W , Van Der Kooij H and Hekman E (2011) A passive exoskeleton with artificial tendons: Design and experimental evaluation. In *2011 IEEE International Conference on Rehabilitation Robotics,* pp. 1–6. 10.1109/ICORR.2011.5975470. 22275668

[r21] Wahl A (1963) Mechanical Springs. Cleveland: Mcgraw-Hill.

[r22] Witte KA , Fiers P , Sheets-Singer AL and Collins SH (2020) Improving the energy economy of human running with powered and unpowered ankle exoskeleton assistance. Science Robotics 5(40), eaay9108. 33022600 10.1126/scirobotics.aay9108

[r23] Yandell MB , Tacca JR and Zelik KE (2019) Design of a low profile, unpowered ankle exoskeleton that fits under clothes: Overcoming practical barriers to widespread societal adoption. IEEE Transactions on Neural Systems and Rehabilitation Engineering 27(4), 712–723. 30872237 10.1109/TNSRE.2019.2904924 PMC6592282

[r24] Yu S , Huang T-HH , Wang D , Lynn B , Sayd D , Silivanov V , Park YS , Tian Y and Su H (2019) Design and control of a high-torque and highly backdrivable hybrid soft exoskeleton for knee injury prevention during squatting. IEEE Robotics and Automation Letters 4(4), 4579–4586.

[r25] Zhang T , Tran M and Huang H (2018) Design and experimental verification of hip exoskeleton with balance capacities for walking assistance. IEEE/ASME Transactions on Mechatronics 23(1), 274–285.

